# Isolation, selection and culture methods to enhance clonogenicity of mouse bone marrow derived mesenchymal stromal cell precursors

**DOI:** 10.1186/s13287-015-0139-5

**Published:** 2015-08-25

**Authors:** Claas Baustian, Shirley Hanley, Rhodri Ceredig

**Affiliations:** Regenerative Medicine Institute, National Centre for Biomedical Engineering Science and School of Medicine, National University of Ireland, Galway, Ireland; Biosciences, National University of Ireland Galway, Newcastle Road, Dangan, Galway, Ireland

## Abstract

**Introduction:**

Conventionally cultured mouse bone marrow mesenchymal stromal cells (mBM-MSC) are a heterogeneous population that often initially contain contaminating haematopoietic cells. Variability in isolation methods, culture protocols and the lack of specific mBM MSC markers might explain this heterogeneity. The aim of this study is to optimise the isolation, culture conditions and selection of mBM-MSC.

**Methods:**

Mouse BM-MSCs were isolated from crushed long bones (cBM) or flushed bone marrow (fBM) from 6–8 week old C57Bl/6 mice. These subpopulations were analysed by flow cytometry using commonly used mBM-MSC cell surface marker, e.g. Sca-1, CD29 and CD44. Cells were cultured and expanded in vitro in hypoxic conditions of either 2 % or 5 % oxygen. Cell sorting and qRT-PCR was used to determine transcript levels of stem cell and lineage related genes in individual subpopulations.

**Results:**

During early passaging not only do contaminating haematopoietic cells disappear, but there is a change in the phenotype of mBM-MSC affecting particularly CD44 and Sca-1 expression. By fluorescence activated cell sorting of CD45^−^/Ter119^−^ mBM stroma based on Sca-1 expression and expansion in hypoxic conditions, we show that Sca-1^+^ cells had higher CFU-F frequencies and showed enhanced proliferation compared with Sca-1^−^ cells. As evaluated by in vitro assays and qRT-PCR, these cells presented in vitro tri-lineage differentiation along osteocyte, chondrocyte, and adipocyte lineages. Finally, by prospective isolation of Sca-1^+^PDGFRα^+^CD90^+^ cells we have isolated mBM-MSC on a single cell level, achieving a CFU-F frequency of 1/4. Functional investigations demonstrated that these MSC clones inhibited T-lymphocyte proliferation.

**Conclusion:**

By positive selection using a combination of antibodies to Sca-1, CD90 and PDGFRα and culturing in hypoxia, we have found a subpopulation of BM cells from C57Bl/6 mice with a CFU-F cloning efficiency of 1/4. To our knowledge these results represent the highest frequencies of mouse MSC cloning from C57Bl/6 mice yet reported.

**Electronic supplementary material:**

The online version of this article (doi:10.1186/s13287-015-0139-5) contains supplementary material, which is available to authorized users.

## Introduction

Mesenchymal stromal cells (MSCs) are used in many research fields and have generated much interest for cell therapies because of their ability to differentiate into various cell types including osteocytes, chondrocytes and adipocytes [1]. While a lot is known about the in-vitro behaviour of mouse and human MSCs, relatively little is known about the in-vivo behaviour of human MSCs. This difference is despite the fact that human MSCs are being used therapeutically in a number of clinical trials. Prospective isolation of both human and mouse MSCs (mMSCs) has been reported but is rarely undertaken. The lack of a reliable method to prospectively isolate mMSCs from bone marrow restricts the use of genetically altered mouse strains to study basic aspects of MSC biology [2]. The aim of this study is to optimise the isolation, culture conditions and selection of mouse bone marrow-derived MSCs (mBM-MSCs).

A key aspect in the investigation of mBM-MSCs is the isolation method employed. Normally, suspensions of bone marrow cells are cultured in plastic dishes with non-adherent cells discarded during passaging. Two common problems associated with this isolation method are, firstly, in early passages there is contamination with adherent haematopoietic cells and, secondly, both mesenchymal and haematopoietic cells in such cultures are heterogeneous [3]. Microscopic examination of the adherent mesenchymal cells show them growing from individual foci, or colonies, and these colonies have been called the colony-forming unit fibroblast (CFU-F) [4]. Difficulties associated with culturing mBM-MSCs as well as mouse strain variations in plating efficiency and the relative ease with which human cells can be cultured have resulted in comparatively more work being done with human MSCs than with mouse-derived MSCs [5, 6]. By culturing adherent cells from both species long term, it became evident that their self-renewal and/or differentiation capacity became impaired [7]. Thus, the MSC-like properties of cells may not be retained after serial passaging in vitro. In order to try and improve the isolation of mBM-MSCs, flow cytometry (FCM) has recently been employed to positively select mBM-MSCs. Several surface markers have been used in these experiments, the most frequent being Stem cell antigen-1 (Sca-1) [8].

Discovered almost 30 years ago as antigens expressed by fetal thymocytes [9], Sca-1 (Ly-6A/E) and stem cell antigen-2 are members of the Ly-6 family of interferon-inducible lymphocyte activation proteins whose genes are located on mouse chromosome 15 [10, 11]. Sca-1 is an 18 kDa mouse glycosylphosphatidylinositol (GPI)-linked cell surface protein and is encoded by the mouse strain-specific *Ly-6A/E* allelic gene [12]. Sca-1 is differentially expressed by lymphocytes from mouse strains differing at the *Ly-6* locus resulting in a 20-fold higher expression in C57Bl/6 mice (Ly-6^b^) compared with BALB/c mice (Ly-6^a^) [13]. In the cell membrane, Sca-1 is associated with protein tyrosine kinases and lipid rafts, suggesting that it may be involved in signal transduction [14, 15]. In C57Bl/6 mice, Sca-1 is a well-established marker of mouse haematopoietic stem cells (HSCs) and in conjunction with additional markers such as CD117 (c-kit) is routinely used for their isolation from bone marrow [16]. Likewise, for mBM-MSC isolation, Sca-1 has been used in conjunction with other markers, but no systematic analysis of Sca-1 expression by cultured mBM-MSCs has so far been reported. Outside the well-characterised haematopoietic system, Sca-1 is expressed by a mixture of stem, progenitor and differentiated cell types in various organs such as bone, bone marrow, muscle, thymus, spleen, kidney and lymph nodes [17]. Sca-1 has been used in combination with other markers to isolate mBM-MSCs [18, 19], and a recent study was able to generate clonal subpopulations of mBM-MSCs by combining Sca-1 and platelet-derived growth factor receptor alpha (PDGFRα) staining which showed tri-lineage differentiation capacity both in vitro and in vivo [20].

Traditionally, mBM-MSCs are grown in vitro in ‘normoxic’ conditions (21 % oxygen). This level of oxygen does not reflect the physiological level in the bone marrow, thereby exposing cells to a higher oxygen concentration than in their native state. Owing to its architecture of medullary sinuses and arteries, the oxygen tension in the bone marrow has been estimated to range from 1 to 7 % [21]. For several stem and progenitor populations, hypoxia is an important factor in stem cell biology, promoting an undifferentiated state [22, 23]. Previous publications have shown that CFU-F frequency, growth and differentiation of mBM-MSCs were negatively affected by normoxic oxygen levels [24–26]. Moreover, expansion of mBM-MSCs in low-oxygen limits the accumulation of chromosomal aberrations, a common problem in mBM-MSC cultures [27]. Despite these negative effects of high-oxygen cultures, low-oxygen culture conditions have not been generally adopted.

In this study we have looked systematically at the evolution of surface markers on mBM-MSCs. We show that markers commonly used for both human and mouse-derived MSC validations, including the mouse marker Sca-1, are upregulated during in-vitro expansion. Cells capable of forming CFU-F were found in both Sca-1^+^ and Sca-1^−^ subpopulations, but these differed in average colony size and culturing cells under hypoxia improved their plating efficiency. In addition, analysis of freshly isolated and cultured Sca-1^+^ and Sca-1^−^ fractions showed significant differences in transcriptomic profiles and differentiation properties. By combining selection for Sca-1^+^ with CD90^+^ and PDGFRα^+^, we obtained a fraction representing 0.02 % of bone marrow which had immunosuppressive properties and a plating efficiency of one in four sorted cells. To our knowledge, this represents one of the highest reported frequencies of CFU-F for mBM-MSCs and will allow a more detailed analysis of mBM-MSC biology.

## Materials and methods

### mBM-MSC isolation

Experimental animals were housed in a specific pathogen-free facility and fed a standard chow diet. All animal procedures were carried out under licence from the Irish Department of Health and Children and were approved by the NUI Galway Animal Care Research Ethics Committee (ID: 12/JULY/02). Femurs and tibias from 6–8-week-old C57Bl/6 mice were dissected and bone marrow flushed with an 18-gauge needle and syringe containing α-MEM + GlutaMAX (Gibco, Paisley, UK). Red blood cell lysis was performed using sterile water for 5 seconds, after which the reaction was quenched using fluorescence-activated cell sorting (FACS) buffer. For the purposes of this manuscript, this cell suspension will be referred to as flushed bone marrow (fBM).

To isolate endosteal lining cells, bone marrow was flushed from femurs and tibias, the bones were crushed with a pestle and mortar, were gently washed with α-MEM + GlutaMAX, and were filtered through a 70 μm cell strainer (Fisher Scientific Waltham, MA, USA). Bone fragments were further cut into 1–2 mm pieces and incubated for 45 minutes at 37 °C in 4 ml α-MEM + GlutaMAX containing 2.5 mg/ml collagenase I (Sigma-Aldrich, St. Louis, USA) and 100 μg/ml DNAse IV (Sigma, St. Louis, USA). Then 10 ml α-MEM + GlutaMAX with 10 % equine serum, 10 % fetal calf serum and 100 units/μg penicillin/streptomycin were added to quench collagenase activity. Finally, the suspension was filtered with a 40 μm cell strainer and centrifuged at 400 × *g* for 5 minutes. For the purposes of this manuscript, this suspension will be referred to as compact bone marrow (cBM).

Cell pellets were resuspended in mMSC medium, FACS buffer (1× phosphate-buffered saline (PBS), 2 % fetal calf serum (FCS), 0.05 % NaN_2_) or FACS sorting buffer (1× Ca and Mg-free PBS (Gibco-Invitrogen, Paisley, UK), 1 % FCS, 25 mM HEPES, 2 mM sodium ethylenediamine tetraacetic acid (Na_2_EDTA)) depending on the intended application.

### mBM-MSC culture conditions

The fBM and cBM derived cell suspensions were plated on 10 mm plates in α-MEM + GlutaMAX supplemented with 10 % equine serum, 10 % fetal calf serum and 100 units/μg penicillin/streptomycin (mMSC medium). Cells were incubated in a humidified incubator at 37 °C supplemented with either 21 %, 5 % or 2 % oxygen. At 3-day or 4-day intervals, the media were changed and non-adherent haematopoietic cells were removed by gentle pipetting. At 10–14 days, when the primary culture was approximately 80 % confluent, cells were detached with trypsin/EDTA for 5 minutes and seeded to new plates. Culture medium was changed every 3–4 days thereafter and subsequent passages were performed when cells reached 60–80 % confluency. Primary articular chondrocytes were prepared from adult C57Bl/6 mice as described previously [28] and were cultured for 10 days prior to RNA extraction.

### Flow cytometry and FACS

Adherent cells were detached with 0.25 % trypsin/0.02 % EDTA, suspended in ice-cold FACS buffer at 10^6^ cells/ml, and incubated for 30 minutes at 4 °C with monoclonal antibodies (listed in Additional file 1). Cells were stained with propidium iodide (PI) (1 μg/ml; Sigma Aldrich) prior to FCM analysis. The FCM analysis was performed using an Accuri® C6 Sampler flow cytometer (Becton Dickinson Biosciences, Erembodegem, Belgium), which was calibrated according to the manufacturer’s recommendations. The BD Accuri® C6 Sampler is a dual-laser cytometer, containing a 488 nm laser and a 640 nm laser and four fluorescence detectors: FL1 (533 ± 30 nm), FL2 (585 ± 40 nm) FL3 (>670 nm) and FL4 (675/42 nm). The BD Accuri® C6 Sampler is a digital cytometer equipped with linear amplifiers and 24-bit digitisation on all detectors. Data are stored and displayed in 16.7 million channels (approximately 7.2 log display). Fluorescence compensation was set using single-stained controls, and matching median compensation algorithms were applied. Data were analysed using CFlowPlus (Becton Dickinson Biosciences) or FlowJo® software (TreeStar Inc., Olten, Switzerland).

Typical gating strategies involved gating on size versus granularity (Forward Scatter (FSC) versus Side Scatter (SSC)), doublet exclusion based on FSC-H versus FSC-A, and dead cell exclusion by gating on cell populations negative for PI fluorescence in FL3. Fluorescent minus one or matched antibody isotype controls were used to define the position of the gates (see Additional file 2).

For FACS, the labelled cells were washed twice, filtered through a 40 μm filter, resuspended in FACS sorting buffer at concentrations of between 10 × 10^6^ and 20 × 10^6^ cells/ml and sorted using a FACSAriaII® sorter (Becton Dickinson Biosciences). Where appropriate, the purity of sorted cell subsets was determined by post-sorting analysis. Additional FACS information can be found in Additional files 3 and 4).

### CFU-F assay

Fluorescence-activated sorted cells isolated from fBM and cBM were plated into a 10 mm plate and incubated for 10 days in a humidified incubator at 37 °C, supplied with 5 % carbon dioxide and either 21 %, 5 % or 2 % oxygen. Subsequently, colonies were fixed and stained with crystal violet. The number of colonies displaying 50 or more cells with spindled mMSC morphology was scored using the Kodak Imager Software (Carestream, Kodak, Rochester, NY, USA) on the Kodak Imager Station 4000MM (Corestream Health Inc., Rochester, NY, USA). Colonies with an area below 1 mm^2^ were scored as ‘small’, and colonies with an area above 1 mm^2^ were scored as ‘large’.

### Differentiation assays

Tri-lineage differentiation capacity was determined using standard chondrogenic, adipogenic and osteogenic differentiation assays [23].

### Quantitative RT-PCR

Total cellular RNA was extracted using TRIzol reagent (Invitrogen, Paisley, UK) according to the manufacturer’s instructions. Reverse transcription reactions were performed with total RNA ≥20 ng/μl using the High Capacity cDNA Reverse Transcript Kit (Applied Biosystems, Life Technologies, Carlsbad, CA, USA), in accordance with the manufacturer’s instructions. Real-time PCR (Lightcycler 480II; Roche, Basel, Switzerland) was performed with 2 μl single-stranded cDNA sample with SYBR Green PCR master mix (Applied Biosystems). The sequences of primers used are shown in Additional file 5. The annealing temperature was 60 °C. Each amplification reaction was checked to confirm the absence of nonspecific PCR product by melting curve analysis. The relative gene expression level was calculated and presented with the 2^–ΔΔCt^ method. Beta-2 microglobulin (β_2_M) was used as an endogenous control to normalise specific gene expression in each sample.

### Mixed lymphocyte reaction assay

cBM MSC clones, derived from single cell sorts, were added in graded numbers to 96-well round-bottom plates and allowed to adhere for 24 hours prior to the addition of mouse splenocytes. The splenocytes were prepared from C57Bl/6 mice as described previously [29]. Cells were labelled using the CellTrace™ CFSE cell proliferation kit as per the manufacturer’s instructions (Molecular Probes®, Life Technologies, Carlsbad, CA, USA). Then 10^5^ CFSE-labelled splenocytes were added to each well, T cells were stimulated with 0.5 μg/ml purified anti-mouse CD3 and CD28 antibodies (Affymetrix eBioscience, Santa Clara, CA, USA) and the co-cultures were incubated for 3 days. After incubation, cells were labelled with anti-mouse CD4-PE and CD8-APC antibodies (Becton Dickinson Biosciences) and T-cell proliferation was examined via flow cytometric analysis.

### Statistical analysis

All experiments were repeated at least three times with independently collected samples and all values are displayed as mean ± standard deviation (SD) unless stated otherwise. Statistical comparisons were analysed with the Student’s *t* test or one-way analysis of variance (ANOVA) (GraphPad Prism v6; GraphPad Software Inc., La Jolla, CA, USA) and *p* <0.05 was considered statistically significant.

## Results

### Effect of culture duration on surface marker expression

**Fig. 1:**
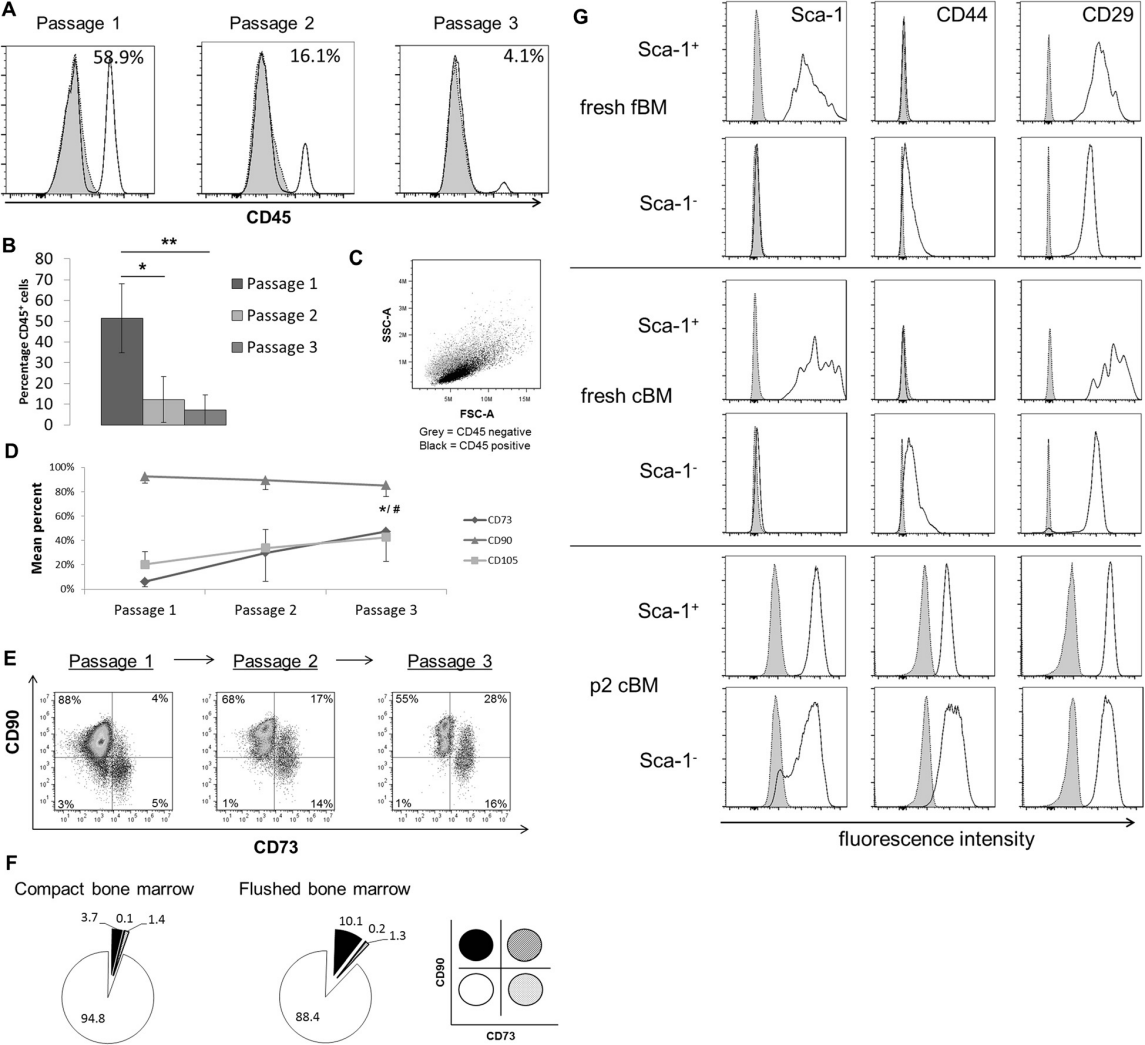
Effect of culture duration on surface marker expression. **a** Flow cytometric analysis of CD45 expression by cBM cultures. Histograms show CD45 staining at the indicated passage numbers with the percent positive cells in each panel (*white, solid line*, CD45; *tinted, dashed line*, isotype control). **b** Summary of CD45 distribution at different passages. Values represent mean ± SD of at least three experiments. Significance testing was done using one-way ANOVA. *p <0.05, **p <0.01. **c** Cytogram display of FSC-A versus SSC-A for different CD45 subpopulations (*grey*, CD45^−^; *black*, CD45^+^). CD45^+^ cells have lower SSC signals than CD45^−^ cells. **d** Human MSC marker expression of gated CD45^−^ cells from cBM during in-vitro passaging. Values represent mean ± SD of at least three experiments. Significance testing was done between passage 1 and passage 3 for CD73 (#), CD90 and CD105 (*). #/*p <0.05, Student’s *t* test. **e** Cytogram displaying the distribution of CD73 versus CD90 over passaging. Quadrants were placed based upon negative control samples in the lower left quadrant. The percent positive cells in each quadrant are shown. **f** Pie-chart representation of the distribution for CD73 versus CD90 of freshly-isolated cBM and fBM. Lower right panel shows the colour codes used to define the four subpopulations expressing CD73 or CD90. **g** Phenotypic evolution of mMSC markers: Sca-1 (*left*), CD44 (*middle*) and CD29 (*right*), in Sca-1 defined subpopulations in freshly-isolated cBM and fBM and cultured cBM preparations. Cultured cBM (*lower panels*) has been expanded in-vitro for two passages. *cBM* compact bone marrow, *fBM* flushed bone marrow

Initial experiments were performed with cells processed from mouse cBM. Cells were cultured under hypoxic (5 % oxygen) conditions and after 7 days were collected and passaged. At this time (passage 1) they exhibited a fibroblast-like morphology typical of mMSCs. When analysed for CD45 expression, mMSC cultures contained >50 % CD45^+^ haematopoietic cells (Fig. 1a), in agreement with previously published reports [30, 31]. Subsequent passaging in hypoxia resulted in a significant reduction in contaminating CD45^+^ cells (Fig. 1a, b).

The relative size (FSC-A) forward scatter area scaling and granularity (SSC-A) side scatter area scaling of the CD45^+^ and CD45^−^ cell populations were compared (Fig. 1c). CD45^+^ and CD45^−^ cells were indistinguishable by FSC-A, but CD45^−^ cells had a consistently higher relative SSC-A value (1.5 ± 0.2) and this ratio remained unchanged with subsequent passaging (see Figure S2A in Additional file 6). Additional phenotypic analysis showed that CD45^+^ cells in these early passage cultures were mostly F4/80^+^CD31^−^ myeloid cells (Figure S2B in Additional file 6).

Flow cytometric analysis was performed on CD45^−^ cells using markers commonly used to characterise human MSCs, namely CD73, CD90 and CD105 [32]. Results obtained showed that there was a significant increase in the percentage of cells expressing CD73, from 6 % at passage 1 to 47 % at passage 3. In addition, the percentage of cells expressing CD105 increased from 20 % to 42 %. CD90 expression decreased slightly from 92 % to 85 %, over three passages (Fig. 1d). Multi-colour flow cytometric analysis of CD73 and CD90 expression allowed further characterisation of the observed phenotypic heterogeneity. As shown in Fig. 1e (left panel), at passage 1 88 % cells were CD90^+^ and CD73^−^ whereas CD90^−^ cells could be subdivided into 60 % CD73^+^ and 40 % CD73^−^ subsets. By passage 3 (Fig. 1e, right panel) the 16 % CD90^−^ cells were mostly CD73^+^ and the CD90^−^CD73^−^ cells (lower left quadrant) had essentially disappeared. CD105 and CD73 exhibited a similar staining progression (data not shown). Thus, as summarised in Fig. 1e, there is clearly a phenotypic evolution of surface marker expression on CD45^−^ cells cultured under hypoxic conditions.

Expression of the same surface markers was analysed on cells freshly isolated from cBM and fBM. As shown in Fig. 1f, most gated CD45^−^/Ter119^−^ cells were CD90^−^CD73^−^ and most of the CD90^+^ cells were CD73^−^.

Figure 1e and f indicates that CD90^−^CD73^−^ cells do not persist under these in-vitro culture conditions.

Sca-1 is a marker frequently used in characterising mMSCs, particularly in C57Bl\6 mice. Given the heterogeneous staining pattern by cultured cells observed, we wished to investigate the phenotypic evolution further. The staining distribution of Sca-1 on both fBM (Fig. 1g, upper panels) and cBM (Fig. 1g, middle panels) was initially quite broad. Although Sca-1^−^ cells remained negative for Sca-1 at passage 1, 90 % expressed this marker by passage 2. At this time, expression of Sca-1 was uniform on both subpopulations (Fig. 1g, lower panels). Surprisingly, for both fBM and cBM, CD44 expression was initially negative on Sca-1^+^ and only weakly expressed by Sca-1^−^ cells (Fig. 1g, middle column). Upon culture, there was an increase in CD44 expression by both subpopulations. CD29 was expressed on both Sca-1^+^ and Sca-1^−^ cells, with a broader staining on freshly-isolated Sca-1^+^ cells (Fig. 1g, upper right panels). After culturing, CD29 was uniformly highly expressed by both subpopulations (Fig. 1g, lower panels).

### Effect of isolation method on surface marker expression and clonogenicity

**Fig. 2:**
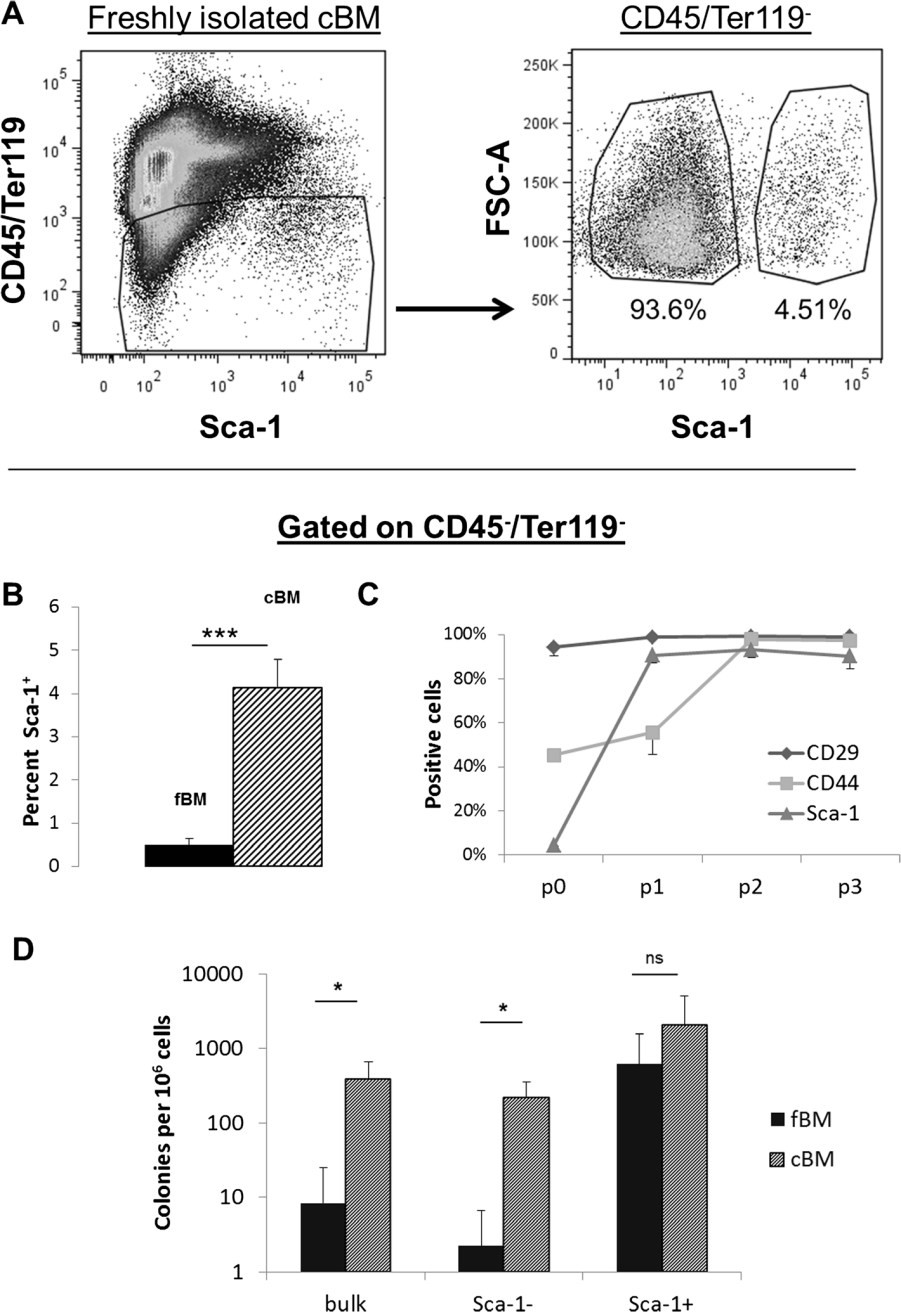
Effect of isolation method on surface marker expression and clonogenicity. **a** Cytograms displaying Sca-1 versus CD45^−^/Ter119^−^ expression in freshly isolated cBM preparations (*left*) and FSC versus Sca-1 of cBM CD45^−^/Ter119^−^ (*right*). **b** Histograms showing a comparison of Sca-1 distribution in fBM versus cBM. Values are mean ± SD of at least three experiments. **c** Evolution of Sca-1, CD44 and CD29 expression on cBM derived CD45^−^ cells in vitro. **d** CFU-F frequencies among Sca-1 sorted subpopulations of fBM versus cBM. Data are the mean ± SD of at least three independent experiments. *p < 0.05, ***p < 0.001, Student’s *t* test. *cBM* compact bone marrow, *fBM* flushed bone marrow, *ns* not significant

The experiments already described were carried out with freshly-isolated cBM. In many publications, fBM is frequently used [33]. Therefore, we wished to compare both isolation methods. As shown in Fig. 2a, 4.5 % of cells processed from cBM and gated on CD45^−^/Ter119^−^ cells were Sca-1^+^. Figure 2b summarises a series of four experiments in which Sca-1 expression on fBM and cBM cells was compared. As shown, compared with fBM, cells isolated from cBM were significantly enriched for Sca-1^+^ cells.

Subsequent experiments focused on the evolution of Sca-1, CD44 and CD29 expression on CD45^−^ cells from cBM and fBM during culture. Sca-1 expression increased from 4 % in fresh cBM to 90 % at passage 3 (Fig. 2c). Cells from fBM were likewise 90 % Sca-1^+^ by passage 3 (results not shown). CD44 expression increased from 45 % in fresh cBM to 97 % at passage 3. CD29 expression was consistently around 95 % (Fig. 2c). Thus, after three passages, cells isolated from cBM were ≥90 % homogeneous for Sca-1, CD44 and CD29 expression yet were distinctly heterogeneous for CD73 (Fig. 1d, e) and for CD105 expression (results not shown). Similar results were obtained from cells isolated from fBM (results not shown).

Next, we wanted to determine the CFU-F frequencies among CD45^−^/Ter119^−^ cells from fBM versus cBM sorted for Sca-1 expression. As shown in Fig. 2d, compared with unsorted (‘bulk’) CD45^−^/Ter119^−^ cells, the CFU-F frequency was always higher among Sca-1^+^ versus Sca-1^−^ FACS sorted cells in both bone marrow preparations. A greater number of CFU-F could thus be recovered from cBM samples, and this was particularly the case for Sca-1^+^ cells (Fig. 2d).

### Effect of oxygen tension on colony counts and size

**Fig. 3:**
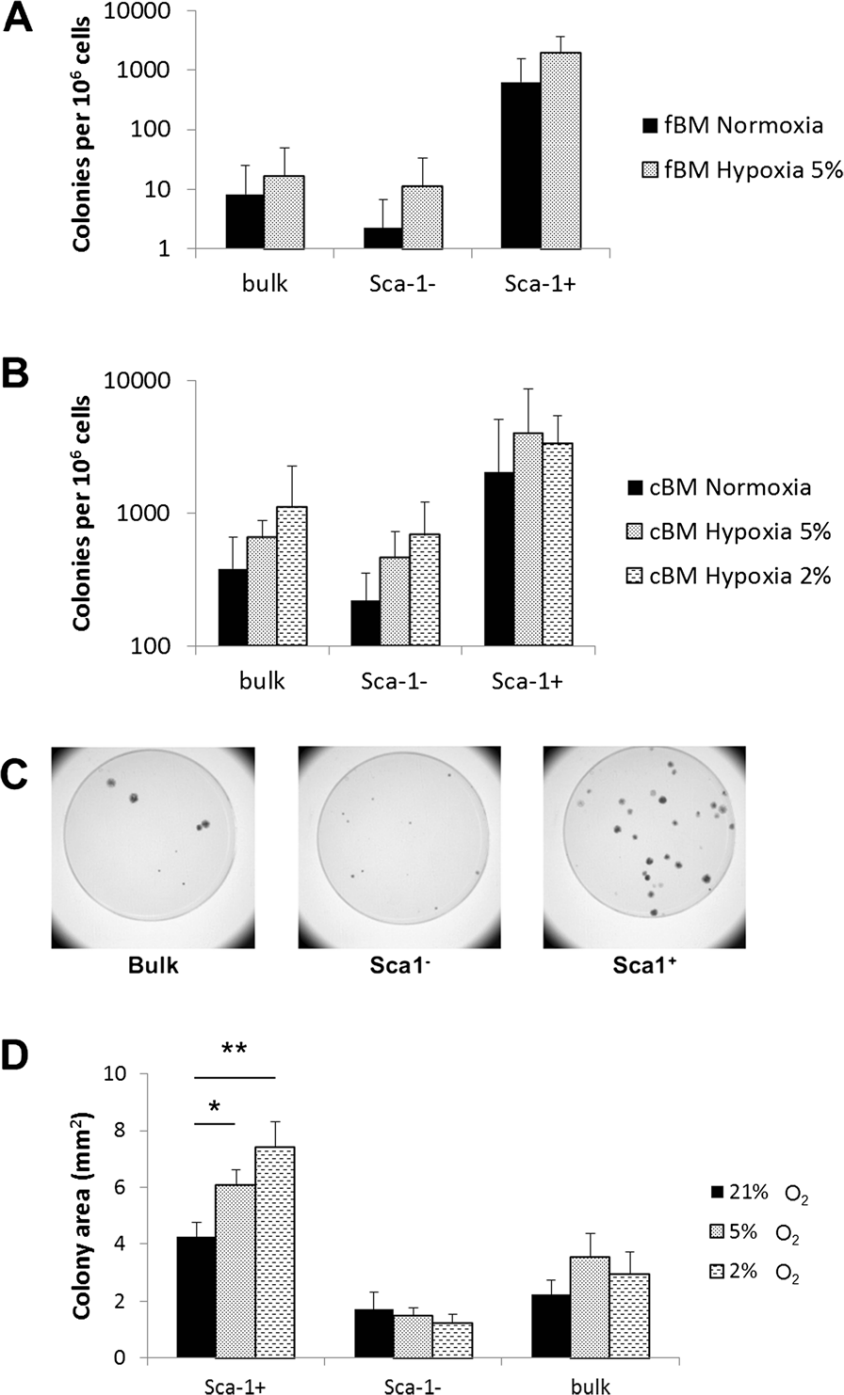
Effect of oxygen tension on colony counts and size. **a** CFU-F frequencies in subpopulations from fBM cultured in 21 % oxygen (normoxia) or 5 % oxygen (hypoxia). **b** CFU-F frequencies in subpopulations from cBM cultured in 21 %, 5 % or 2 % oxygen. Data are the mean ± SD of at least three independent experiments, one-way ANOVA. **c** Colonies from the indicated populations of sorted cBM. ‘Bulk’ refers to unseparated cells. **d** Summary of colony size for the different subpopulations in 21 %, 5 % or 2 % oxygen. Data are the means ± standard error of the mean of >100 colonies from at least three independent experiments. *p <0.05, **p <0.01, one-way ANOVA. *cBM* compact bone marrow, *fBM* flushed bone marrow, *O*_*2*_ oxygen

Recently, hypoxia has been used to improve the in-vitro growth of MSCs [23]. We therefore compared the CFU-F frequencies of cells cultured under normoxic (21 % oxygen) versus hypoxic (5 % oxygen) conditions. For fBM, hypoxia improved CFU-F frequencies in both bulk and Sca-1^−^ subpopulations (Fig. 3a). Given that the overall CFU-F frequencies were higher among cBM, we decided to compare two hypoxic concentrations (2 % and 5 % oxygen) on cells from cBM. As shown in Fig. 3b there was a slight increase in CFU-F correlated with the degree of hypoxia for the bulk and Sca-1^−^ cells, but the difference between 2 % and 5 % oxygen was not statistically significant.

As already mentioned (Fig. 2d), CFU-F frequencies and recovery were higher among cBM and therefore cBM was used for all subsequent experiments. When counting CFU-F, we realised there were size differences between colonies. Indeed whereas ‘bulk’ CD45^−^/Ter119^−^ cells showed heterogeneity in CFU-F colony size, there was a dramatic difference between Sca-1^−^ (small colonies) and Sca-1^+^ (large colonies) sorted subpopulations (Fig. 3c). To quantify this difference, we used the Kodak Imager 4000 software to measure the sizes of individual colonies. Raw data of the size distribution of >100 colonies from four independent experiments are summarised in Fig. 3d. The mean colony diameter of Sca-1^+^ cells is increased with the degree of hypoxia (Fig. 3d). However, Sca-1^−^ cells seem to be little or not affected by oxygen levels. As shown by FSC-A histograms (Figure S3A in Additional file 7), freshly isolated Sca-1^+^ cells are significantly larger than their Sca-1^−^ counterparts. During in-vitro expansion, these differences lose significance (Figure S3A, B in Additional file 7). Morphologically, CFU-F formed by Sca-1^+^ and Sca-1^−^ cells showed differences in phenotype, with Sca-1^−^ cells exhibiting a small, cobblestone-like phenotype versus a larger, fibroblast-like phenotype for Sca-1^+^ cells (see Figure S3C in Additional file 7).

### Use of Sca-1 as a selection marker for MSC isolation

**Fig. 4:**
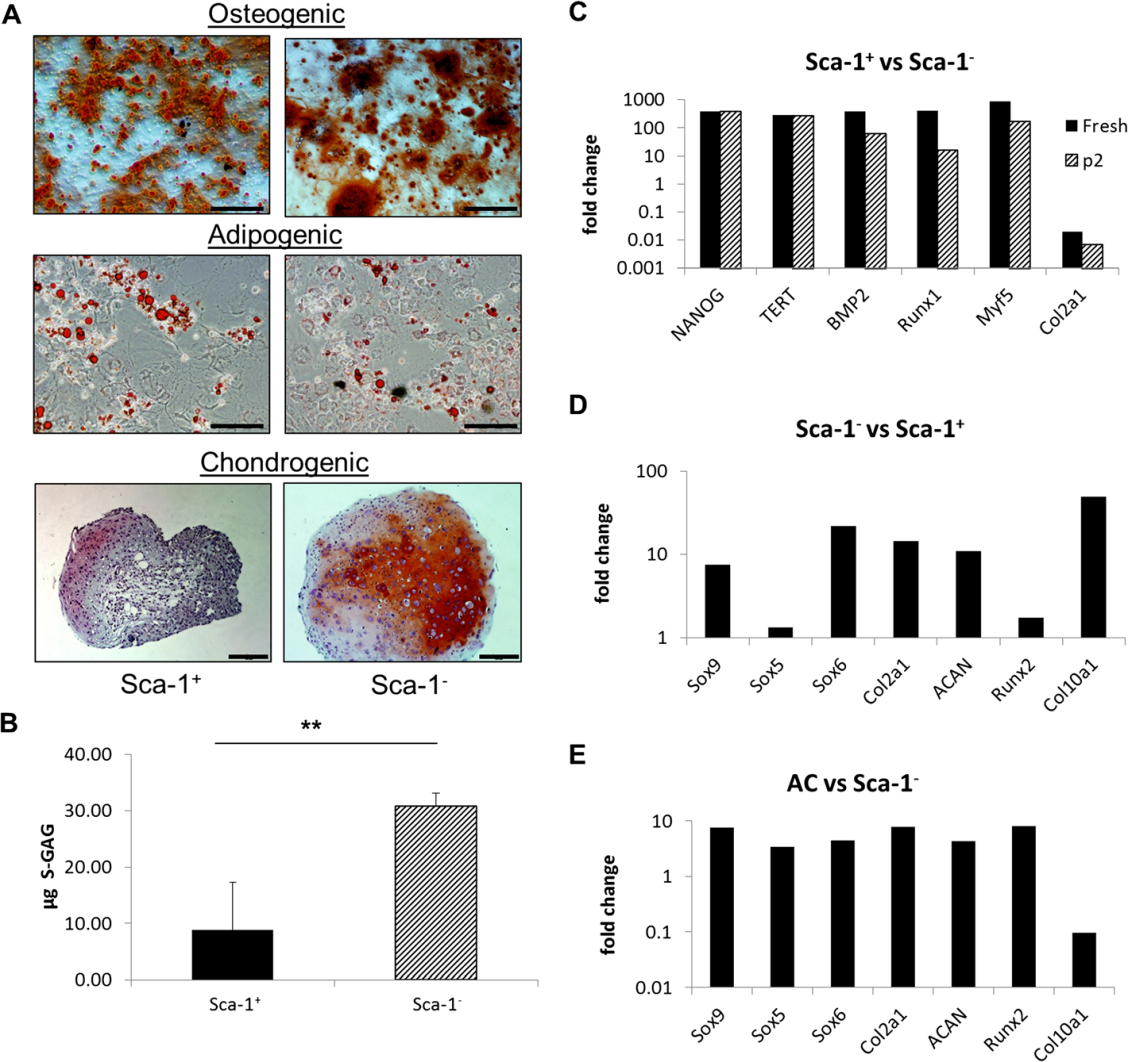
Use of Sca-1 as a selection marker for MSCs. **a** Sca-1^+^ and sca-1^−^ from cBM were cultured in osteogenic (*upper*), adipogenic (*middle*) and chondrogenic (*lower*) differentiation media followed by Alizarin Red S (*upper*), Oil Red O (*middle*) or Safranin O (*lower*) staining. Osteogenic and adipogenic images, bar = 100 μm; chondrogenic images, bar = 200 μm. **b** Chondrogenic pellet cultures from the indicated subpopulations assayed for S-GAG content. **c** Fold change of Sca-1^+^ relative to Sca-1^−^ expression of stem and lineage-specific gene transcripts in freshly isolated and cultured (passage 2 (p2)) cells. Fold change of relative expression of transcripts associated with chondrogenesis in **d** freshly isolated Sca-1^−^ versus Sca-1^+^ sorted cells and **e** cultured articular chondrocytes versus cultured Sca-1^−^ cells. Data are the mean ± SD of at least three independent experiments. **p <0.01, Student’s *t* test. *s-GAG* sulfated glycosaminoglycans

Next we examined the differentiation capacity of Sca-1^+^ and Sca-1^−^ cells to the osteocyte, adipocyte and chondrocyte lineages. After two passages, cells were transferred to the corresponding differentiation conditions, and after a further 14 days cells were analysed for osteocyte and adipocyte differentiation; chondrocyte differentiation was measured after 21 days. As shown in Fig. 4a, both Sca-1^+^ and Sca-1^−^ cells differentiated along the osteocyte and adipocyte lineages; however, Sca-1^−^ cells showed increased differentiation to chondrocytes as demonstrated by Safranin-O staining for proteoglycans (Fig. 4a, lower panel). This was confirmed by quantification for sulfated glycosaminoglycans (S-GAG) with an approximately threefold greater S-GAG content in Sca-1^−^ cells (Fig. 4b). No significant difference between the two subpopulations in osteogenic or adipogenic differentiation was found (data not shown).

To address whether cells freshly isolated from cBM were likewise chondrocyte ‘primed’, we investigated expression of transcripts characteristic of stem cells (NANOG, TERT), osteocytes (BMP2), myogenic/adipocytes (Myf5) and chondrocytes (Col2a1) in both Sca-1 subpopulations by quantitative RT-PCR. In these experiments, transcripts for β_2_M served as endogenous control. In freshly isolated cBM cells, expression of transcripts for NANOG, TERT, BMP2 and Myf5 were considerably higher (500-fold to 800-fold) among Sca-1^+^ cells relative to Sca-1^−^ cells (Fig. 4c). In contrast, expression of Col2a1, a chondrocyte-specific gene, was considerably downregulated among Sca-1^+^ relative to Sca-1^−^ cells (50-fold reduction in expression). Upon culture in hypoxia (passage 2), the expression of NANOG and TERT remained unchanged, the expression of BMP2, Runx1 and Myf5 decreased (30-fold to 300-fold) and Col2a1 was further downregulated. This indicated a higher expression of Col2a1 in the Sca-1^−^ population in both freshly isolated and cultured cells (Fig. 4c).

To investigate further the chondrogenic capacity of Sca-1^−^ cells, we looked in more detail at transcripts for genes specific for chondrocyte differentiation. Transcripts of the chondrogenic genes ACAN, Sox9, Col2a1 and Sox6 were at least fivefold higher in Sca-1^−^ relative to Sca-1^+^ cells with the greatest difference observed in Col10a1 expression (Fig. 4d). Minimal differences were observed in Runx2 and Sox5 expression levels.

To compare the relative levels of chondrocyte-specific genes in freshly isolated Sca-1^−^ cells versus mature chondrocytes, primary chondrocytes were isolated from the knees of adult C57Bl/6 mice and cultured for 10 days in 5 % hypoxia prior to RNA extraction. Primary articular chondrocytes generally showed a higher expression for all chondrogenic transcripts relative to Sca-1^−^ cells (Fig. 4e), with differences ranging from approximately fourfold for Sox5, Sox6 and ACAN to approximately eightfold for Sox9, Col2a1 and Runx2. The exception was Col10a, where expression was 11-fold less in articular chondrocytes versus freshly isolated Sca-1^−^ cells (Fig. 4e).

### PDGFRα^+^/CD90^+^ subpopulation shows an increased CFU-F frequency

**Fig. 5:**
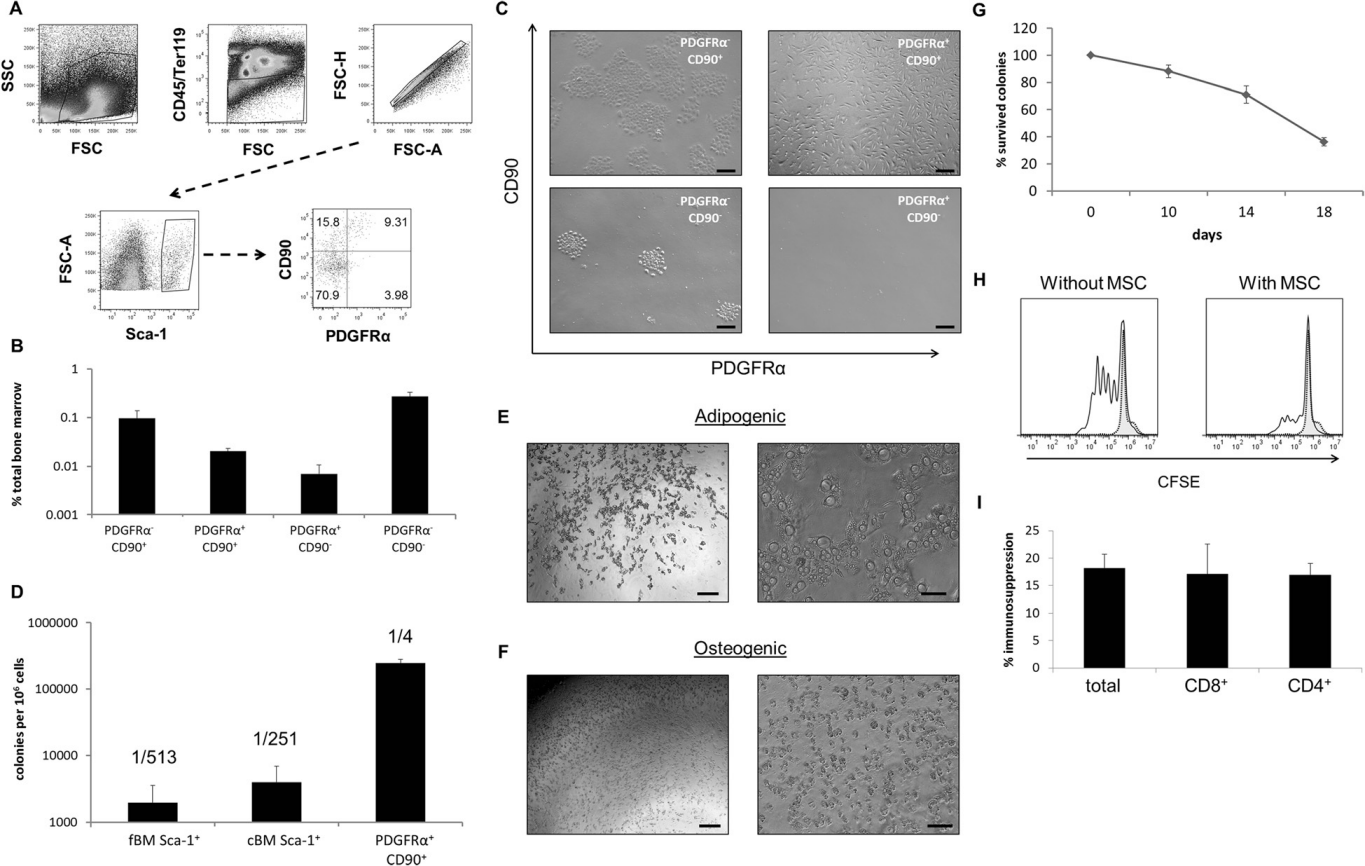
Clonal isolation of the PDGFRα^+^/CD90^+^ subpopulation. **a** Gating strategy for the single-cell isolation of PDGFRα^+^/CD90^+^ cells. Cells were initially gated on FSC and SSC followed by CD45^−^/Ter119^−^ cells (*upper*). Gated Sca-1^+^ cells among CD45^−^/Ter119^−^ cells (*lower left*) are then subdivided into the four CD90/PDGFRα subpopulations (*lower right*) where percentages in each quadrant are indicated. **b** Distribution of subpopulations in fresh cBM. **c** Bright-field images of expanded cells of the indicated subpopulation after 10 days of culturing. **d** CFU-F frequencies of Sca-1^+^ cells in fBM (*left*), cBM (*middle*) and PDGFRα^+^CD90^+^Sca-1^+^ cBM (*right*) cells. Expanded PDGFRα^+^/CD90^+^ cells after 7 days in **e** adipogenic and **f** osteogenic differentiation media. First column = 4× magnification (image bar = 200 μm) and second column = 10× magnification (image bar = 100 μm). **g** Survival curve for PDGFRα^+^/CD90^+^ colonies during in-vitro expansion. **h** Representative histogram of CFSE staining among gated T cells of CD3/CD28 stimulated splenocytes with and without addition of cloned PDGFRα^+^/CD90^+^ cells (*white, solid line*, stimulated splenocytes; *tinted, dashed line*, unstimulated splenocytes). Bar = 200 μm. **i** Percentage immunosuppression among different subsets of T lymphocytes. *cBM* compact bone marrow, *fBM* flushed bone marrow, *MSC* mesenchymal stromal cell, *PDGFRα* platelet-derived growth factor receptor alpha

Based on the results, Sca-1^+^ cells were clearly enriched in transcripts for the stem cell genes NANOG and TERT (Fig. 4c) and showed the greater CFU-F yield. We therefore decided to further subdivide Sca-1^+^ cells. PDGFRα in conjunction with Sca-1 has been used previously to purify mMSCs from cBM on a single cell level with a reported CFU-F frequency of 1/22.5 [20]. To further increase the CFU-F frequency of freshly single-cell sorted mouse bone marrow, we additionally stained cells with CD90, a marker used for the characterisation of human MSCs and consistently expressed by freshly-isolated mMSCs (Fig. 1d). We subdivided the CD45^−^/Ter119^−^/Sca-1^+^ population into four distinct subpopulations of PDGFRα/CD90-expressing cells (Fig. 5a). The distribution of these subpopulations in freshly harvested bone marrow was similar in fBM and cBM (data not shown). The percentages of bone marrow cells expressing these surface markers for cBM are shown in Fig. 5b. When expanded in vitro, PDGFRα^+^/CD90^+^ from cBM showed a spindle-shaped, fibroblastic morphology typical for MSCs (Fig. 5c), whereas the PDGFRα^−^/CD90^−^ and PDGFRα^−^/CD90^+^ cell populations generated cobblestone-like morphologies. When compared with unfractionated Sca-1^+^ cells, the CFU-F frequency in fBM and cBM was 1/513 and 1/251 respectively (Fig. 5d). Single-cell sorted PDGFRα^+^/CD90^+^/Sca-1^+^ from cBM yielded a CFU-F frequency of 1/4.

Following their initial isolation and identification, individual clones were expanded for several days and passaged at 10, 14 and 18 days. During this time not all clones continued growing, and by 18 days approximately 40 % of initially-plated clones survived (Fig. 5g). Surviving clones were divided and exposed to media for osteogenic and adipogenic differentiation, but unfortunately none of the cells survived after 14 or 21 days of in-vitro differentiation. After 7 days of in-vitro differentiation, lipid vacuoles could be seen in osteogenic and adipogenic differentiating cultures (Fig. 5e, f).

In control medium, clones were tested for their ability to inhibit the proliferation of CD3/CD28-stimulated T cells. All 12 clones tested in this assay showed potent inhibitory activity on T-cell proliferation at an MSC:T-cell ratio of 1:100. A result from a representative clone is shown in Fig. 5h. Additional staining with CD4 and CD8 monoclonal antibodies revealed a simultaneous degree of inhibition of proliferation in both T-lymphocyte subsets mediated by cloned PDGFRα^+^/CD90^+^ cells (Fig. 5i).

## Discussion

In this study, we have used flow cytometry to analyse the evolution of surface markers on cultured mMSCs and to use a combination of markers to prospectively isolate, with high efficiency, clonal populations that had immune regulatory properties. This approach will enable the use of genetically modified mouse strains to further investigate the basic biology of MSCs. Similar experiments could be done with human cells in order to improve and harmonise human MSC preparations. Until recently [20, 34], most experiments with mMSCs have been conducted with unsorted, uncloned cells—so-called ‘bulk’ MSCs. As shown in Fig. 1, without sorting ‘bulk’ MSCs are heterogeneous, containing myeloid cells at early passages, and are a phenotypically evolving collection of cells. Our phenotypic analysis of CD45^−^/TERE119^−^ bone marrow cells showed a change in composition of cells expressing the human MSC markers CD73, CD90 and CD105 (Fig. 1d, e) and the mMSC markers CD44 and Sca-1 (Fig. 1g).

Human MSCs have been studied intensively and are already used in clinical trials [35, 36]. CD73, CD90 and CD105, among others, are routinely used for the definition of human MSCs [32]. Unfortunately these markers are not MSC specific and, as reviewed recently [33], expression varies on passaging. mMSCs also exhibit heterogeneous staining for these markers, making their use in prospective isolation methods problematic [37]. Among CD45^−^/Ter119^−^ fresh bone marrow cells, CD73^−^/CD90^−^ cells represent a major population, but only a minor population (3 %) in passage 1 expanded cells (Fig. 1e). The low percentage of these cells in early passages suggests that they do not survive well in vitro. As reported previously [8], CD73 is barely detected on freshly isolated cells, but is highly expressed on cultured cells. The slow increase over time of CD73 expression (Fig. 1d, e) and reports on its upregulation in hypoxia [38] support the assumption of marker upregulation upon culturing. CD90 (Thy-1) was originally a prototypic T-cell marker but was later found to be expressed on human MSCs. However, its expression on cells from C57Bl/6 mice is controversial, with groups claiming its absence [5, 6] or its presence [18, 20] on mMSCs. In our experience, CD90 can be used as a reliable marker for the prospective isolation of mMSCs (Fig. 5).

Additional analysis showed that expression of both CD44 and Sca-1 antigens increased upon culturing. This is somewhat expected given that expression of both antigens can be regulated upon cell signalling [10, 39]. CD44 is well established as an activation marker on mouse and human T cells [39]. CD44 is used frequently as a marker to define mMSCs [40, 41], yet recent research has shown that CD44 might not be a useful marker for the isolation of MSCs because MSCs could be isolated from sorted CD44^−^ cells and because CD44 was acquired in vitro [41]. We showed herein that gated CD45^−^/Sca-1^+^ fresh fBM and cBM cells were also CD44^−^ and that CD44 was acquired in vitro (Fig. 1g). Additional experiments focused on Sca-1 expression, showing that Sca-1 was acquired by sorted Sca-1^−^ cells (Fig. 1G) [6, 19, 42]. Given that the possible contamination of sorted Sca-1^−^ cells by Sca-1^+^ was at most 2 %, and the kinetics of Sca-1^+^ cell growth, the simplest interpretation of these results is that Sca-1 was acquired by cultured Sca-1^−^ cells rather than being the result of outgrowth from Sca-1^+^ contaminants.

Currently, two major isolation methods are used to isolate mMSCs from bone marrow. The traditional method consists of flushing out the bone marrow (fBM) and using mechanical sheer force to obtain single-cell suspensions. This may leave behind potential stem/progenitor cells residing at the endosteum. By collagenase digestion of bone fragments (cBM), endosteum residing cells can be harvested and purified using flow cytometry. A general conclusion from our studies was that the CFU-F frequency was considerably higher among such cBM cells (Fig. 2b). There is general agreement that MSCs in the bone marrow, and the HSCs which they support, can be found at different anatomical locations that differ also in oxygen availability (reviewed in [22]). Many such MSC/HSC niches are found close to cortical bone in a relatively hypoxic environment and therefore, for maximum CFU-F recovery, crushing bones followed by collagenase digestion is clearly advantageous.

Surprisingly, when CD45^−^/Ter119^−^ bone marrow cells from either fBM or cBM were sorted based upon Sca-1 expression, the CFU-F colony size varied significantly between subpopulations (Fig. 3c, d). Although differences in CFU-F colony size have been reported previously [43], to our knowledge this is the first report indicating differences in colony size among freshly isolated MSC subpopulations sorted based upon surface marker expression. Although Sca-1^+^ cells were slightly larger by FSC-A than their Sca-1^−^ counterparts (Additional file 7), cell size alone is unlikely to explain differences in colony size. Additionally, hypoxia had an effect both on CFU-F frequency (Fig. 3b) and average colony size (Fig. 3d). This was particularly the case for Sca-1^+^ cells (Fig. 3d). This could imply that the majority of Sca-1^+^ cells are located close to the endosteum where oxygen levels are low [22]. In summary, hypoxia improved CFU-F frequency among Sca-1^−^ cells (Fig. 3b) but had little effect on cell growth (Fig. 3d). In contrast, hypoxia did not improve CFU-F frequency among Sca-1^+^ cells but did improve their growth. The improvement in both CFU-F frequency and cell growth of bulk MSCs in hypoxia is readily explained by the fact that ‘bulk’ MSCs contain a mixture of Sca-1^+^ and Sca-1^−^ cells. The poor chondrogenic differentiation capacity of ‘bulk’ mMSCs might also be a result of the low CFU-F frequency of the more chondrogenic Sca-1^−^ population.

Taken together, the smaller size of colonies, the lower CFU-F frequency and the lack of a significant response of Sca-1^−^ cells to hypoxia may suggest they reside in different anatomical locations and represent less differentiated cells. In analogy with haematopoiesis, differences in mean colony size could indicate different stem/progenitor subpopulations [44]. Slowly proliferating stem cells would thus form smaller colonies appearing later in culture than rapidly proliferating progenitors forming early, larger colonies. However, our transcriptomic analysis of freshly isolated Sca-1^+^ and Sca-1^−^ cells indicated the opposite, with higher levels of NANOG and TERT expression among Sca-1^+^ cells (Fig. 4c). We have shown that both Sca-1^+^ and Sca-1^−^ cells have the capacity to give rise to osteoblasts, chondrocytes and adipocytes (Fig. 4a). In addition, and confirming results from differentiation assays (Fig. 4a), the Sca-1^−^ subpopulation appeared to express increased levels of transcripts characteristic of chondrogenesis (Fig. 4d). The Sca-1^−^ in-vitro differentiation to chondrocytes, as measured by GAG accumulation (Fig. 4b), was thus superior to that of Sca-1^+^ cells. Indeed, in a recent publication, Chan et al. [45] identified a similar pro-chondrogenic progenitor in mouse bone marrow. Interestingly, it has been shown that Sca-1^−/−^ mice develop age-related osteoporosis owing to reduced numbers of osteoprogenitors and osteoblasts and display a weakened bone structure and bone material caused by the reduced number of MSC; this also resulted in impaired adipogenesis in vitro [46].

In the seminal paper by Morikawa et al. [20], cells sorted from cBM with the CD45^−^, Ter119^−^, Sca-1^+^ and PDGFRα^+^ (so-called PαS markers) phenotype had a CFU-F frequency of 1/22.5. Individual colonies were isolated and some had in-vitro tri-lineage differentiation potential even following re-isolation from in vivo, indicating the existence of true mesenchymal stem cells. We have directly sorted single cells from bone marrow and, in addition to PDGFRα, have used CD90 expression as a selection marker. As shown in Fig. 5b, this combination of markers allows the isolation of CFU-F with a frequency of 1/4 among PDGFRα^+^/CD90^+^ cells. This represents a higher frequency than previously reported by Morikawa et al., most probably because CD90^−^/PDGFRα^+^ cells, comprising about 1/3 Sca-1^+^/PDGFRα^+^ cells, failed to grow in vitro (Fig. 5c, lower right). In addition to the increase in CFU-F frequencies, morphological differences between subpopulations of sorted cells were also evident (Fig. 5c). The full implication of these differences in cell morphology requires additional investigation beyond the scope of this manuscript.

Despite an initial high plating efficiency of sorted Sca-1^+^/PDGFRα^+^/CD90^+^ cells, their continued in-vitro growth was limited (Fig. 5g), a finding reminiscent of mouse T-cell cloning experiments. This was particularly evident when CFU-F clones were transferred to differentiation medium. Recently, dramatic changes in behaviour of clonal populations of mMSCs have been reported [47]. The authors suggest that isolation of individual cells from within a bulk population may alter the cell’s genetic programme. Despite our failure to show complete tri-lineage differentiation of clones, in control cultures some clones continued to expand, allowing their immunosuppressive properties to be analysed. As shown in Fig. 5h, i, such clones strongly inhibited the in-vitro division of activated T cells.

## Conclusion

We have recently shown that mMSCs respond to hypoxia by upregulating transcripts for stem cell genes resulting in improved growth and differentiation [23]. We have taken advantage of these results to prospectively isolate and clone MSCs from C57Bl/6 bone marrow. By positive selection using a combination of antibodies to Sca-1, CD90 and PDGFRα and culturing in hypoxia, we have found a subpopulation of CD45^−^/Ter119^−^ bone marrow cells from C57Bl/6 mice with a CFU-F cloning efficiency of 1/4. Individual clones suppressed T-cell proliferation, thereby revealing immunosuppressive properties. To our knowledge, these results represent the highest frequencies of mMSC cloning from C57Bl/6 mice so far reported.

## Additional files

Additional file 1: Table S1. Presenting a summary of monoclonal antibodies used in the experiments. (XLSX 9 kb) Additional file 2: Figure S1. Showing the representative gating strategy for flow cytometric analysis. Cells were gated based on their size and granularity (*upper left*) and doublets were excluded based on FSC-A versus FSC-H (*upper right*). CD45^+^ and non-viable (PI^+^) cells were gated out (*lower left*) and quadrant gates were drawn based on fluorescent minus one controls containing matched antibody isotypes (*lower right*). (TIFF 231 kb) Additional file 3:
**A document presenting the methods for FACS and gelatine coating.** For FACS, cells isolated from fBM or cBM were resuspended in ice-cold FACS buffer and stained for 30 minutes at 4 °C in the dark with previously optimised concentrations of monoclonal antibodies listed in Additional file 1. The labelled cells were washed twice, filtered through a 40 μm filter, resuspended in FACS sorting buffer at concentrations between 10 × 10^6^ and 20 × 10^6^ cells/ml and sorted using a BD FACSAriaII® sorter fitted with a 70 μm nozzle for fBM and an 85 μm nozzle for cBM derived cell suspensions The BD FACSAriaII® optical configuration and corresponding fluorochomes/dyes used is defined in Additional file 4. SYTOX® blue dead cell stain (Life Technologies) was used for dead cell exclusion and added just prior to the sort (Additional file 2). Cell doublets were excluded based on FSC-A versus FSC-H and fluorescence minus one controls were used to define the position of the gates. Where appropriate, the purity of sorted cell subsets was determined with purities >95 % observed. FACS single cells were seeded on plastic or gelatine-coated 96 well plates. For gelatine coating, 96-well flat-bottom plates were coated with 0.1 % gelatine and incubated for 2 hours at room temperature. Gelatine was removed and the vessels were incubated for 1 hour at 37 °C. Gelatine-coated vessels were used immediately or stored at 4 °C in Dulbecco’s PBS for a maximum of 2 weeks. (DOCX 81 kb) Additional file 4: Table S2. Presenting the optical configuration and corresponding fluorochomes/dyes of the BD FACSAriaII® cell sorter. (XLSX 9 kb) Additional file 5: Table S3. Presenting the sequences of quantitative RT-PCR primers used in the experiments. (XLSX 9 kb) Additional file 6: Figure S2. Showing characterisation of CD45 hematopoietic cells. **A** Ratio of SSC-A between CD45^−^ and CD45^+^ cells. **B** Co-expression of CD45 and F4/80 (*black*, CD45 only; *grey*, CD45^+^ F4/80). Data are the mean ± SD of at least three independent experiments. (TIFF 61 kb) Additional file 7: Figure S3 Showing the size and colony appearance of Sca-1 subpopulations. **A** Size differences in freshly isolated Sca-1 subpopulations from cBM. **B** Size differences in cultured Sca-1 subpopulations. Bright-field images of **C** Sca-1^−^ and **D** Sca-1^+^ expanded colonies after 10 days of culture. First column = 4× magnification (bar = 200 μm) and second column = 10× magnification (bar = 100 μm). Data are the mean ± SD of at least three independent experiments. **p* <0.05, Student’s *t* test. (TIFF 1643 kb)

## Abbreviations

ANOVA: Analysis of variance
β2M: Beta-2 microglobulin
cBM: Compact bone marrow
CFU-F: Colony-forming unit fibroblast
EDTA: Ethylenediamine tetraacetic acid
FACS: Fluorescence-activated cell sorting
fBM: Flushed bone marrow
FCM: Flow cytometry
FCS: Fetal calf serum
FSC: Forward Scatter
GPI: Glycosylphosphatidylinositol
HSC: Haematopoietic stem cell
mBM-MSC: Mouse bone marrow mesenchymal stromal cell
mMSC: Mouse mesenchymal stromal cell
MSC: Mesenchymal stromal cell
PBS: Phosphate-buffered saline
PDGFRα: Platelet-derived growth factor receptor alpha
PI: Propidium iodide
Sca-1: Stem cell antigen-1
SD: standard deviation
S-GAG: Sulfated glycosaminoglycans
SSC: Side Scatter
