# Supplementary figures and images for: Isolation, selection and culture methods to enhance clonogenicity of mouse bone marrow derived mesenchymal stromal cell precursors
Source: Stem Cell Res Ther. 2015 Aug 25;6(1):151. doi: 10.1186/s13287-015-0139-5 (PMC4549076; doi:10.1186/s13287-015-0139-5)

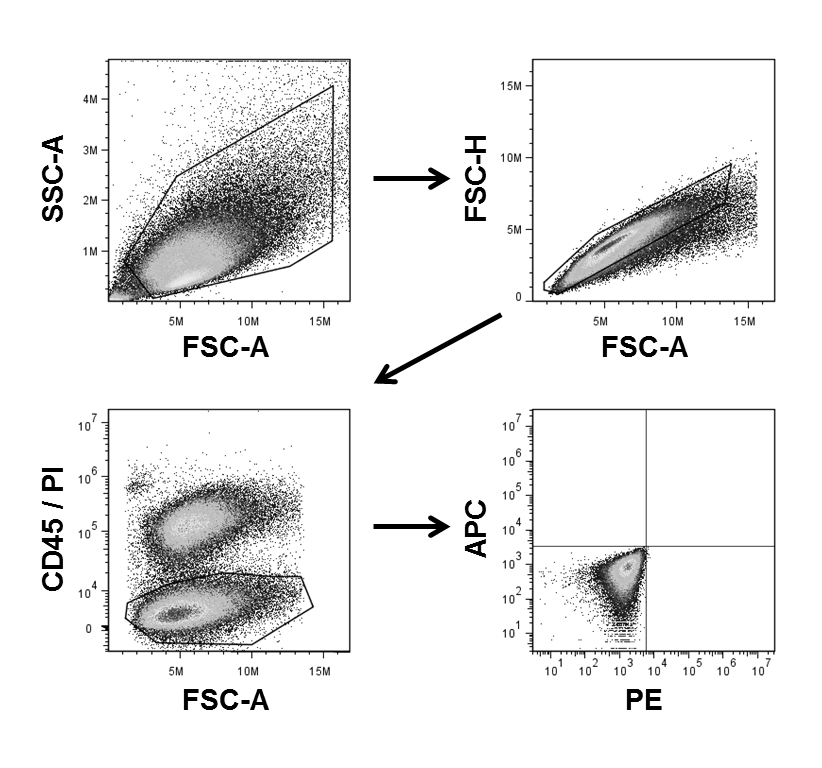

Supplement: Additional file 2: Figure S1. — Showing the representative gating strategy for flow cytometric analysis. Cells were gated based on their size and granularity (upper left) and doublets were excluded based on FSC-A versus FSC-H (upper right). CD45+ and non-viable (PI+) cells were gated out (lower left) and quadrant gates were drawn based on fluorescent minus one controls containing matched antibody isotypes (lower right). (TIFF 231 kb) [file 13287_2015_139_MOESM2_ESM.tif]

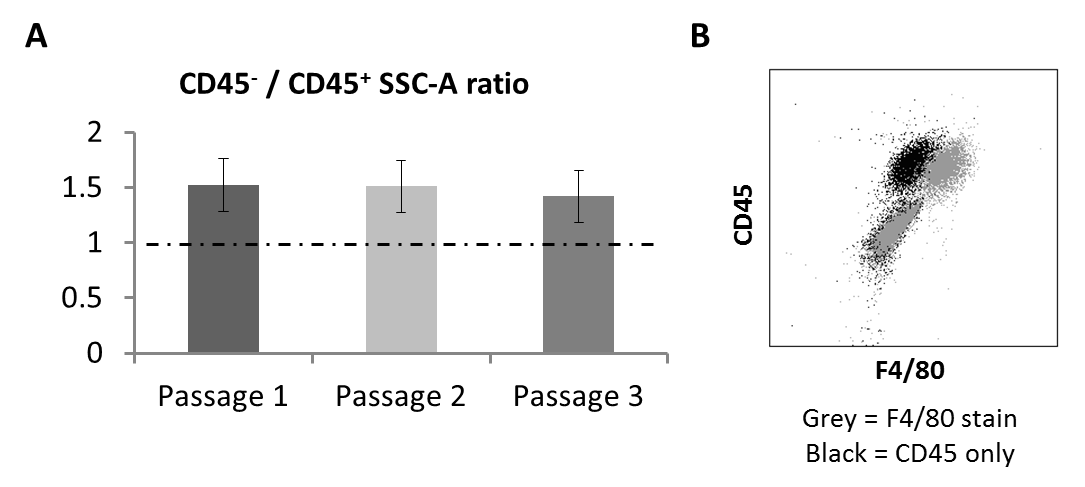

Supplement: Additional file 6: Figure S2. — Showing characterisation of CD45 hematopoietic cells. A Ratio of SSC-A between CD45− and CD45+ cells. B Co-expression of CD45 and F4/80 (black, CD45 only; grey, CD45+ F4/80). Data are the mean ± SD of at least three independent experiments. (TIFF 61 kb) [file 13287_2015_139_MOESM6_ESM.tif]

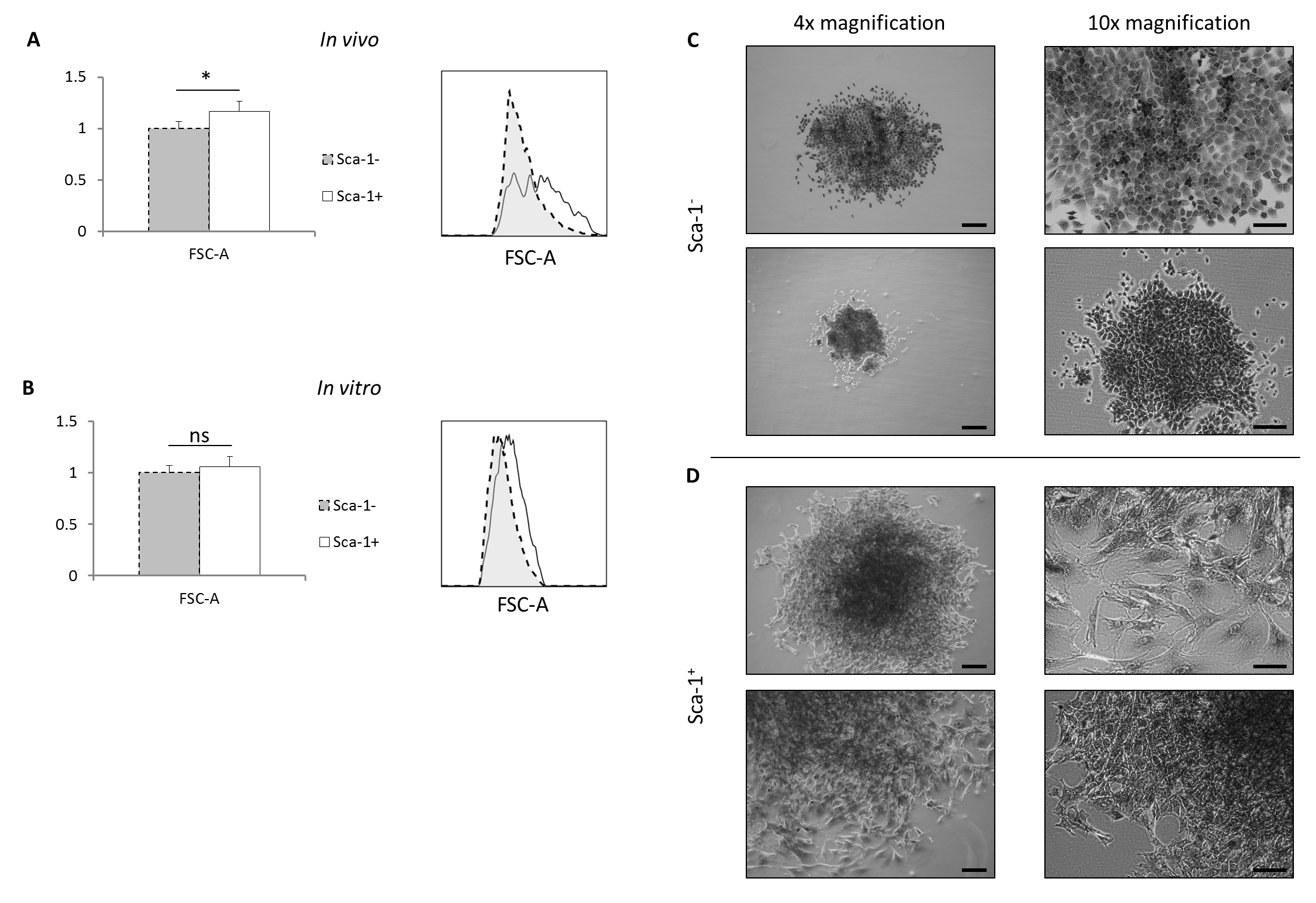

Supplement: Additional file 7: Figure S3 — Showing the size and colony appearance of Sca-1 subpopulations. A Size differences in freshly isolated Sca-1 subpopulations from cBM. B Size differences in cultured Sca-1 subpopulations. Bright-field images of C Sca-1− and D Sca-1+ expanded colonies after 10 days of culture. First column = 4× magnification (bar = 200 μm) and second column = 10× magnification (bar = 100 μm). Data are the mean ± SD of at least three independent experiments. *p <0.05, Student’s t test. (TIFF 1643 kb) [file 13287_2015_139_MOESM7_ESM.tif]
