# Supplementary material for: Isolation, selection and culture methods to enhance clonogenicity of mouse bone marrow derived mesenchymal stromal cell precursors
Source: Stem Cell Res Ther. 2015 Aug 25;6(1):151. doi: 10.1186/s13287-015-0139-5 (PMC4549076; doi:10.1186/s13287-015-0139-5)
Supplement: Additional file 3: — A document presenting the methods for FACS and gelatine coating. For FACS, cells isolated from fBM or cBM were resuspended in ice-cold FACS buffer and stained for 30 minutes at 4 °C in the dark with previously optimised concentrations of monoclonal antibodies listed in Additional file 1. The labelled cells were washed twice, filtered through a 40 μm filter, resuspended in FACS sorting buffer at concentrations between 10 × 106 and 20 × 106 cells/ml and sorted using a BD FACSAriaII® sorter fitted with a 70 μm nozzle for fBM and an 85 μm nozzle for cBM derived cell suspensions The BD FACSAriaII® optical configuration and corresponding fluorochomes/dyes used is defined in Additional file 4. SYTOX® blue dead cell stain (Life Technologies) was used for dead cell exclusion and added just prior to the sort (Additional file 2). Cell doublets were excluded based on FSC-A versus FSC-H and fluorescence minus one controls were used to define the position of the gates. Where appropriate, the purity of sorted cell subsets was determined with purities >95 % observed. FACS single cells were seeded on plastic or gelatine-coated 96 well plates. For gelatine coating, 96-well flat-bottom plates were coated with 0.1 % gelatine and incubated for 2 hours at room temperature. Gelatine was removed and the vessels were incubated for 1 hour at 37 °C. Gelatine-coated vessels were used immediately or stored at 4 °C in Dulbecco’s PBS for a maximum of 2 weeks. (DOCX 81 kb) [file 13287_2015_139_MOESM3_ESM.docx]

**Supplementary methods.**

**Fluorescence activated cell sorting (FACS)**

Cells isolated from fBM or cBM were re-suspended in ice-cold FACS Buffer and stained for 30 min at 4°C in the dark with previously optimised concentrations of monoclonal antibodiess listed in Supplementary Table 1. The labelled cells were washed twice, filtered through 40μm filter, re-suspended in FACS sorting buffer at concentrations between 10-20x10^6^ cells/ml and sorted using a BD FACSAriaII® sorter fitted with a 70µm nozzle for fBM and an 85µm nozzle for cBM derived cell suspensions The BD FACSAriaII® optical configuration and corresponding fluorochomes/dyes used is defined in Supplementary Table 2. SYTOX® blue dead cell stain (Life Technologies) blue was used for dead cell exclusion and added just prior to the sort (Supplementary Fig. 1). Cell doublets were excluded based on FSC-A vs FSC-H and fluorescence minus one controls were used to define the position of the gates. Where appropriate, the purity of sorted cell subsets was determined and with purities > 95% observed. FACS single cells were seeded on plastic or gelatine-coated 96 well plates.

**Gelatine coating**

96-well flat bottom plates were coated with 0.1% gelatine and incubated for 2 hours at room temperature. Gelatine was removed and the vessels were incubated for 1 hour at 37°C. Gelatine coated vessels were used immediately or stored at 4°C in Dulbecco’s PBS for a maximum of 2 weeks.

**Supplementary Figure 1.** **Representative gating strategy for flow cytometric analysis.** Cells were gated based on their size and granularity (upper left cytogram) and doublets were excluded based on FSC-A vs FSC-H (upper right cytogram).CD45^+^ and non-viable (PI^+^) cells were gated out (lower left cytogram) and quadrant gate were drawn based on fluorescent minus one controls containing matched antibody isotypes (lower right cytogram).

**Supplementary Figure 2. Characterisation of CD45 hematopoietic cells.**

(A) Ratio of SSC-A between CD45^-^ and CD45^+^ cells. (B) Co-expression of CD45 and F4/80 (black, CD45 only; grey, CD45 ^+^ F4/80). Data are the means ± SD of at least three independent experiments.

**Supplementary Figure 3. Size and colony appearance of Sca-1 subpopulations.**

(A) Size differences in freshly isolated Sca-1 subpopulations from cBM. (B) Size differences in cultured Sca-1 subpopulations. (C) Brightfield images of Sca-1^-^ and (D) Sca-1^+^ expanded colonies after 10d of culture. First column = 4x magnification (bar = 200μm) and second column 10x magnification (bar = 100μm). Data are the means ± SD of at least three independent experiments. *p < 0.05, Student’s *t* test.
